# Association of serum leptin and ghrelin with depressive symptoms in a Japanese working population: a cross-sectional study

**DOI:** 10.1186/1471-244X-14-203

**Published:** 2014-07-30

**Authors:** Shamima Akter, Ngoc Minh Pham, Akiko Nanri, Kayo Kurotani, Keisuke Kuwahara, Felice N Jacka, Kazuki Yasuda, Masao Sato, Tetsuya Mizoue

**Affiliations:** Department of Epidemiology and Prevention, Center for Clinical Sciences, National Center for Global Health and Medicine, Toyama 1-21-1, 162-8655 Shinjuku-ku, Tokyo, Japan; IMPACT Strategic Research Centre, Deakin University, Melbourne, Australia; Department of Metabolic Disorder, Diabetes Research Center, National Center for Global Health and Medicine, Shinjuku-ku, Tokyo, Japan; Department of Applied Biological Chemistry, Graduate School of Bioresource and Bioenvironmental Sciences, Kyushu University, Fukuoka, Japan

**Keywords:** Depressive symptoms, Serum leptin, Serum ghrelin, Japanese

## Abstract

**Background:**

Leptin and ghrelin have been implicated in the pathogenesis of major depression. However, evidence is lacking among apparently healthy people. This study examined the relationship of these appetite hormones to depressive symptoms in a Japanese working population.

**Methods:**

A cross-sectional study was conducted in 2009 among 497 Japanese employees (287 men and 210 women) aged 20–68 years. Fasting serum leptin and ghrelin levels were measured using a Luminex suspension bead-based multiplexed array. Depressive symptoms were assessed using the Center for Epidemiologic Studies Depression (CES-D) scale. Logistic regression analysis was performed to estimate odds ratio (OR) and 95% confidence interval (CI) for depressive symptoms with adjustment for potential confounders.

**Results:**

The prevalence of depressive symptoms (CES-D ≥16) was 26.5% and 33.3% among men and women, respectively. Women in the middle and highest tertiles of leptin levels showed lower odds for depressive symptoms compared with those in the lowest level, although the trend association was not statistically significant (*P*_*trend*_ = 0.14). Higher ghrelin levels were associated with increased odds for depressive symptoms in women (*P*_*trend*_ = 0.02). The multivariable adjusted OR (95% CI) of having depressive symptoms for the lowest through highest tertiles of ghrelin levels were 1.00 (reference), 1.71 (0.76 - 3.86), and 2.69 (1.16 - 6.28), respectively. Neither leptin nor ghrelin was associated with depressive symptoms in men.

**Conclusions:**

Results suggest that lower leptin and higher ghrelin levels may be related to higher prevalence of depressive status among Japanese women.

## Background

Depression is an important public health issue, affecting approximately 350 million people worldwide, and is a significant contributor to the global burden of disease [1, 2]. It is an illness that impairs an individual’s ability to function at work or cope with daily life [3, 4]. At its worst, depression can lead to suicide, which is responsible for the loss of approximately one million lives every year [2]. In Japan, the number of patients identified with depression has been increasing [5] and Japan has one of the world’s highest suicide rates [6]. New insights into the pathogenesis of depression to support the development of preventive strategies for this illness are therefore essential.

Leptin, an anorexigenic hormone produced by adipose tissue, regulates food intake and body weight [7, 8], while ghrelin, an orexigenic hormone produced mainly in the stomach, also plays a role in regulating appetite, weight and body fat [8, 9]. These hormones have opposite effects, not only on regulating appetite and body weight, but also on mood and stress pathways. In animal models of depression, administration of leptin has been shown to improve depressive behaviors and exert antidepressant-like effects [10, 11]. Similarly, a potential role of ghrelin in the regulation of stress and anxiety is suggested [12]. Acute stress increases ghrelin secretion in rats [13] and central ghrelin administration increases anxiety and depressive-like behaviours [14].

Appetite and food intake are often altered in patients with major depression. Although weight loss has been recognized as a typical sign of depression, weight gain has also been observed [15]. Chronic stress or atypical depression may cause weight gain through increased ingestion of calorie dense foods [16]. In addition, some anti-depressive drugs are known to induce weight gain [17]. Given important roles of leptin and ghrelin in the regulation of appetite and weight, depressive status as well as medication for depression may also influence the levels of these hormones.

Several neural pathways linking leptin and ghrelin to mental disorders have been suggested. Leptin may have a role in modulating monoamine neurotransmission [18, 19]. Ob/ob mice lacking leptin had lower dopamine release and decreased concentrations of tyrosine hydroxylase, the rate limiting enzyme in dopamine synthesis, in the nucleas accumbens, and these effects were reversed by leptin treatment [18]. Hyperactivity of the hypothalamic-pituitary-adrenal (HPA) axis is a common feature of depression [20] and accumulating evidence suggests that leptin reduces HPA activity [21, 22]. Neurotrophic actions of leptin may be another mechanism explaining the relationship with depressive disorders. In animal studies, leptin has been shown to reduce depressive behaviors through the upregulation of brain-derived neurotropic factor in the hippocampus [23]. In contrast, ghrelin suppresses serotonin neurotransmission [24] and increases HPA axis activity [25, 26]. Ghrelin’s suppressive effect on the central release of monoamines and stimulatory action on the HPA axis suggest that rising ghrelin may contribute to the mechanisms responsible for the development of depression.

Several studies in humans have examined the association between leptin and depression, but their results are inconsistent. Some clinical studies have reported lower levels of leptin among depressed individuals [27–31] and suicide attempters [32], while others have found either no difference [33] or even higher blood levels of leptin in depressed individuals [34–36], as compared with healthy controls. Inconsistent findings have also been observed regarding the association between ghrelin levels and depression [27, 30, 37–39] and suicide attempters [40]. The results of these clinical studies may reflect changes of weight and appetite as a result of depression [15, 33]. Only two studies, to our knowledge, have investigated the relationship between circulating leptin levels and depression in the general population: one in women only [41] and another in elderly men and women [42]. Moreover, although some data suggest racial differences in blood levels of leptin and ghrelin [43, 44], studies on this issue have been limited to Westerners. Similarly, given a large difference in blood leptin and ghrelin levels between men and women [31, 37, 42], there may be a sex difference in the association between these hormones and depression. Here, we examined the association of serum leptin and ghrelin levels and depressive symptoms among Japanese men and women.

## Methods

### Study procedure and subjects

This study was conducted in July and November 2009 among employees of two municipal offices in northeastern Kyushu, Japan, as described elsewhere [45]. At the time of the periodic health examination, all full time workers (n = 605), except those on long sick or maternity leave, were invited to participate in this study. Participants were asked to fill in the survey questionnaires beforehand, which were checked by research staff for completeness and, where necessary, clarified by questioning the subjects during the examination. Data were also obtained from routine health checkup, including anthropometric measurements, biochemical data, and information about medical history, smoking, and alcohol drinking. Blood and urine specimens were also collected. The Ethics Committee of the National Center for Global Health and Medicine, Japan, approved the study and written informed consent was obtained from all subjects before the survey.

Of 605 eligible employees, 567 subjects (325 men and 242 women) aged 20–68 years participated in the survey (response rate 94%). We excluded 32 subjects with a history of diabetes, cardiovascular disease, or cancer. We further excluded 22 subjects without leptin and ghrelin measurement and 25 subjects whose blood sample was not obtained in fasting status. Some subjects fell into more than one exclusion criteria. Finally, 497 subjects (287 men and 210 women) remained for this study.

### Depressive symptoms

Depressive symptoms were assessed with the Japanese version [46] of the Center for Epidemiologic Studies Depression (CES-D) scale [47]. This scale consists of 20 questions addressing 6 symptoms of depression experienced by participants during the preceding week: depressed mood, feelings of guilt or worthlessness, helplessness or hopelessness, psychomotor retardation, loss of appetite, and sleep disorders. Each question is scored on a scale of 0–3 according to the frequency of the symptom, and the total score ranges from 0 to 60. The criterion validity of the CES-D scale has been well established both in Western [47] and Japanese [46] subjects. Depressive symptoms were defined as present when subjects had a CES-D score of ≥16. Another cutoff value for the definition of depressive symptoms (CES-D ≥ 19) that may be more suitable for Japanese was also used [48].

### Blood measurements

Blood samples were obtained after an overnight fast mostly in the morning between 8:30 and 12:00 a.m. Venus blood (7 mL) was drawn in tube (without addition of protease inhibitors) and then taken in a cooler box to a laboratory. The blood was centrifuged for 10 minutes and was divided into a maximum of six tubes (0.5 mL each) at –80°C or –20°C until analysis. About 15 μL of serum was used to analyze leptin and ghrelin. To quantify serum concentrations of leptin and ghrelin, a Luminex suspension bead-based multiplexed array was performed using Bio-Plex 3D suspension array system and Bio-Plex Pro human diabetes assay panel (Bio-Rad Laboratories, Hercules, CA). The intra-assay coefficients of variation were 11% for leptin and 5% for ghrelin.

### Other variables

Body height was measured to the nearest 0.1 cm with the subjects standing without shoes. Body weight in light clothes was measured to the nearest 0.1 kg. Body mass index (BMI) was calculated as the body weight (kg) divided by the square of the body height (m^2^). Smoking status, alcohol drinking, marital status, job title, job position, and non-occupational physical activity were self-reported in the lifestyle questionnaire. Job title was used to create categories for occupational physical activity; sedentary work included managerial and clerical jobs, whereas physically active work included child-care, school lunch cooking, and technical jobs at water, sewer, and waste treatment facility. Non-occupational physical activity was expressed as daily minutes spent for walking or cycling during commute to or from work and weekly hours engaged in each of five different activities (walking, low-, moderate-, and high-intensity activities, and gardening). The sum of time spent for all the non-occupational activities were expressed in hours per week.

### Statistical analyses

Because the association of leptin and ghrelin with depressive symptoms may not be linear, we used tertile categories of leptin and ghrelin. Characteristics of study subjects across tertiles of leptin or ghrelin level and their trend association were assessed using linear regression analysis for continuous variables or Mantel-Haenszel chi-square test for categorical variables. Serum leptin and ghrelin levels were considerably higher in women than men, thus we present the data separately. Multiple logistic regression analyses were used to estimate odds ratios (ORs) and 95% confidence intervals (CIs) of depressive symptoms for the tertiles of leptin and ghrelin levels, taking the lowest tertile as a reference. We adjusted for a range of potential confounding factors, which were a priori selected with reference to reviews on risk factor for depression [49, 50] as well as our previous works on folate and ferritin [51, 52] . The two participating offices in the present study are denoted as workplace A and workplace B. In the first model, we adjusted for age (year, continuous), and workplace (A or B). In the second model, we further adjusted for marital status (married or unmarried), job position (low, medium, or high), occupational physical activity (active or sedentary), non-occupational physical activity (0, >0 to <2 hours/week, or ≥2 hours/week), current smoking (yes or no), alcohol drinking (yes or no), total energy intake (kcal/day, continuous), BMI (kg/m^2^, continuous), logarithmic serum ferritin (μg/L, continuous), and logarithmic serum folate (ng/mL, continuous). Trend association was assessed by assigning ordinal numbers 1–3 to tertiles of leptin and ghrelin levels. The effect modification by sex on the association between leptin, ghrelin, and depressive symptoms was assessed using the likelihood ratio test in the full adjusted model. Two-sided *P* values <0.05 were regarded as statistically significant. All analyses were performed using statistical software Stata version 12.1 (StataCorp, College Station, Texas USA).

## Results

The prevalence of depressive symptoms was 26.5% and 33.3% among men and women, respectively using the standard cutoff value (CES-D ≥ 16). Mean values of serum leptin and ghrelin levels were significantly higher in women than men (mean leptin: men, 1.88 ng/mL, women, 3.45 ng/mL; mean ghrelin: men, 0.59 ng/mL, women, 0.82 ng/mL) (*P* <0.001 for both). In men, the median and range of leptin (ng/mL) from lowest through highest tertiles were 0.56 (0.11 - 1.03), 1.36 (1.04 - 1.96), and 3.16 (1.97 - 10.64). The corresponding values (ng/mL) in women were 0.96 (0.08 - 1.95), 2.95 (1.96 - 3.89), and 5.65 (3.90 - 16.55). With regard to ghrelin, median and range (ng/mL) from lowest through highest tertile were 0.32 (0.12 - 0.41), 0.54 (0.42 - 0.72), and 0.89 (0.73 - 2.84) in men, and were 0.41(0.17 - 0.63), 0.78 (0.64 - 0.92), and 1.20 (0.93 - 3.21) in women.

Subjects’ characteristics according to tertiles of leptin in men and women are presented in Table 1. Men with higher leptin levels were more likely to be older and had higher BMI, but were less likely to be in a low job position. In women, participants with higher leptin levels had higher BMI and had a higher energy intake. Subjects’ characteristics across tertiles of ghrelin in men and women are presented in Table 2. In both men and women, subjects with higher ghrelin had lower BMI, but were more likely to be an alcohol drinker.

**Table 1:** **Characteristics of study subjects according to tertiles (T) of leptin (ng/mL)**

	Men				Women			
	T1 (low)	T2	T3 (high)	Trend ***P*** ^***a***^	T1 (low)	T2	T3 (high)	Trend ***P*** ^***a***^
n	96	96	95		70	70	70	
Age (mean ± S.D., year)	42.7 ± 10.6	45.6 ± 11.3	45.8 ± 11.1	0.049	42.3 ± 10.1	41.4 ± 10.1	44.4 ± 10.2	0.24
BMI (mean ± S.D., kg/m^2^)	21.1 ± 2.3	22.8 ± 1.9	25.8 ± 3.2	<0.01	19.4 ± 2.8	20.9 ± 2.0	23.2 ± 2.8	< 0.01
Workplace (A, %)^b^	28.1	35.4	32.6	0.50	44.3	11.4	8.6	< 0.01
Married (%)	75.0	84.4	80.0	0.39	72.9	54.3	72.9	0.98
Job position (low, %)	59.4	51.0	44.2	0.04	71.4	64.3	58.6	0.11
Sedentary work (%)	89.6	86.5	93.7	0.35	64.3	74.3	67.1	0.72
Non-job physical activity (≥2 h/week, %)^c^	51.0	42.7	47.4	0.61	24.3	24.3	28.6	0.56
Current smoker (%)	65.6	57.3	55.8	0.17	4.3	7.1	1.4	0.41
Current alcohol drinker (%)	69.8	70.8	68.4	0.84	45.7	50.0	48.6	0.74
Energy intake (kcal/day)	1921 ± 539	1864 ± 439	1983 ± 580	0.42	1509 ± 383	1562 ± 411	1679 ± 426	0.01
Serum folate (ng/mL) median (IQR)	5.2 (4.3-7.3)	5.5 (4.3-6.7)	5.2 (4.1-6.6)	0.41	6.6 (5.6-8.5)	6.3 (4.9-8.8)	6.3 (5.7-7.6)	0.29
Serum ferritin (μg/L) median (IQR)	123.5 (86.0-184.0)	149.0 (96.6-224.0)	172.0 (105.0-251.0)	0.31	36.1 (12.7-81.4)	39.9 (15.8-77.6)	36.1 (14.1-85.7)	0.98

Abbreviation: *BMI* body mass index, *IQR* interquartile range.

^a^On the basis of Mantel-Haenszel chi-square test for categorical variables and linear regression analysis for continuous variables, assigning ordinal numbers 1–3 to tertiles of leptin.

^b^Two worksites of our study population was denoted as workplace A and workplace B.

^c^Physical activity was expressed as the sum of weekly hours spent for sport activity, as well as walking and cycling on commuting to and from work.

**Table 2:** **Characteristics of study subjects according to tertiles (T) of ghrelin (ng/mL)**

	Men				Women			
	T1 (low)	T2	T3 (high)	Trend ***P*** ^***a***^	T1 (low)	T2	T3 (high)	Trend ***P*** ^***a***^
n	96	96	95		70	70	70	
Age (mean ± S.D., year)	47.0 ± 11.2	43.2 ± 10.6	43.9 ± 11.1	0.052	44.6 ± 9.9	42.5 ± 10.9	41.0 ± 9.4	0.24
BMI (mean ± S.D., kg/m^2^)	24.3 ± 3.5	23.2 ± 2.9	22.2 ± 2.7	<0.01	22.2 ± 2.9	21.2 ± 2.8	20.1 ± 2.9	< 0.01
Workplace (A, %)^b^	25.0	31.3	40.0	0.08	24.3	28.6	11.4	0.06
Married (%)	78.1	79.2	82.1	0.49	68.6	64.3	67.1	0.86
Job position (low, %)	50.0	50.0	54.7	0.51	51.4	74.3	68.6	0.03
Sedentary work (%)	84.4	93.8	91.6	0.09	68.6	65.7	71.4	0.72
Non-job physical activity (≥2 h/week, %)^c^	55.2	32.3	53.7	0.60	24.3	25.7	27.1	0.56
Current smoker (%)	39.6	40.6	41.1	0.84	2.7	1.4	8.6	0.09
Current alcohol drinker (%)	64.6	66.7	77.9	0.046	37.1	51.4	55.7	0.03
Energy intake (kcal/day)	1939 ± 585	1897 ± 534	1933 ± 444	0.93	1622 ± 420	1561 ± 432	1567 ± 385	0.43
Serum folate (ng/mL) median (IQR)	5.3 (4.3 - 6.8)	5.3 (4.3 - 6.7)	5.2 (3.9 - 6.8)	0.43	6.4 (5.4 - 7.3)	6.4 (5.5 - 8.8)	6.5 (5.5 - 8.8)	0.07
Serum ferritin (μg/L) median (IQR)	136.0 (84.7 - 189.5)	153.5 (101.6 - 245.0)	147.0 (90.2 - 211.0)	0.61	28.6 (9.6 - 60.2)	38.6 (16.7 - 89.9)	42.1 (16.8 - 81.4)	0.48

Abbreviation: *BMI* body mass index, *IQR* interquartile range.

^a^On the basis of Mantel-Haenszel chi-square test for categorical variables and linear regression analysis for continuous variables, assigning ordinal numbers 1–3 to tertiles of ghrelin.

^b^Two worksites of our study population was denoted as workplace A and workplace B.

^c^Physical activity was expressed as the sum of weekly hours spent for sport activity, as well as walking and cycling on commuting to and from work.

The odds ratios for depressive symptoms according to tertiles of leptin and ghrelin are presented in Table 3. Women in the middle and highest tertiles of leptin levels showed lower odds for depressive symptoms compared with those in the lowest level, although the trend association was not statistically significant (*P*_*trend*_ = 0.14). In the multivariable adjusted model, compared with women in the first tertile of leptin, those in the second and third tertile had 59% (OR 0.41, 95% CI 0.17-0.97) and 53% (OR 0.47, 95% CI 0.18-1.23) lower odds for depressive symptoms, respectively. A similar association was observed between leptin and depressive symptoms, when the presence of depressive symptoms was defined as CES-D ≥ 19. In women, the multivariable adjusted ORs (95% CIs) for depressive symptoms in first, second, and third tertiles of leptin were 1.00 (reference), 0.34 (0.13-0.92), and 0.51 (0.18-1.47), respectively (*P*_*trend*_ = 0.16). In men, there was no association between leptin levels and depressive symptoms using the two different cut off values of depressive symptoms.

**Table 3:** **Odds ratios and 95% confidence intervals for depressive symptoms according to tertiles of leptin and ghrelin**

	Men				Women				P for interaction^g^
	T1 (low)	T2	T3 (high)	Trend ***P*** ^a^	T1 (low)	T2	T3 (high)	Trend ***P*** ^a^	
**Leptin (ng/mL)**									
Median serum leptin (ng/mL)	0.56	1.36	3.16		0.96	2.95	5.65		
CES-D (15/16)									
With^b^/without depressive symptoms^c^ (n)	26/70	23/73	27/68		27/43	20/50	23/47		
Age- and workplace-adjusted OR (95% CI)	1.00	0.89 (0.46 - 1.71)	1.12 (0.59 - 2.12)	0.73	1.00	0.55 (0.26 - 1.16)	0.64 (0.30 - 1.36)	0.27	
Multivariable-adjusted OR (95% CI)^d^	1.00	0.98 (0.48 - 1.99)	1.33 (0.58 - 3.09)	0.52	1.00	0.41 (0.17 - 0.97)	0.47 (0.18 - 1.23)	0.14	0.18
CES-D (18/19)									
With^e^/without depressive symptoms^f^ (n)	20/76	15/81	19/75		20/50	13/57	17/53		
Age- and workplace-adjusted OR (95% CI)	1.00	0.72 (0.34 - 1.51)	0.97 (0.47 - 1.96)	0.92	1.00	0.51 (0.22 - 1.19)	0.75 (0.34 - 1.69)	0.35	
Multivariable-adjusted OR (95% CI)^d^	1.00	0.83 (0.37 - 1.87)	1.28 (0.50 - 3.25)	0.64	1.00	0.34 (0.13 - 0.92)	0.51 (0.18 - 1.47)	0.16	0.21
**Ghrelin (ng/mL)**									
Median serum ghrelin (ng/mL)	0.32	0.54	0.89		0.41	0.78	1.20		
CES-D (15/16)									
With^b^/without depressive symptoms^c^ (n)	28/68	25/71	23/72		20/50	22/48	28/42		
Age- and workplace-adjusted OR (95% CI)	1.00	0.83 (0.44 - 1.58)	0.78 (0.40 - 1.50)	0.45	1.00	1.19 (0.57 - 2.46)	1.72 (0.84 - 3.55)	0.14	
Multivariable-adjusted OR (95% CI)^d^	1.00	0.68 (0.34 - 1.38)	0.74 (0.36 - 1.51)	0.40	1.00	1.71 (0.76 - 3.86)	2.69 (1.16 - 6.28)	0.02	0.15
CES-D (18/19)									
With^e^/without depressive symptoms^f^ (n)	20/76	16/80	19/76		17/53	17/53	19/51		
Age- and workplace-adjusted OR (95% CI)	1.00	0.62 (0.29 - 1.29)	0.74 (0.36 - 1.53)	0.41	1.00	1.13 (0.50 - 2.55)	1.35 (0.60 - 3.08)	0.25	
Multivariable-adjusted OR (95% CI)^d^	1.00	0.47 (0.21 - 1.06)	0.79 (0.37 - 1.70)	0.52	1.00	1.45 (0.60 - 3.49)	2.76 (1.71 - 5.37)	0.03	0.22

Abbreviation: *OR* odds ratio, *CI* confidence interval.

^a^Based on multiple logistic regression analysis, with ordinal numbers 1–3 assigned to tertile categories of leptin and ghrelin.

^b^Defined as a Center for Epidemiologic Studies Depression Scale score of ≥16.

^c^Defined as a Center for Epidemiologic Studies Depression Scale score of <16.

^d^Adjusted for age (year, continuous), workplace (A or B), marital status (married or unmarried), job position (low, medium, or high), occupational physical activity (active or sedentary), non-occupational physical activity (0, >0 to <2 hours/week, or ≥2 hours/week), current smoking (yes or no), alcohol drinking (yes or no), total energy intake (kcal/day), body mass index (kg/m^2^, continuous), logarithmic serum folate (ng/mL, continuous), and logarithmic serum ferritin (μg/L, continuous).

^e^Defined as a Center for Epidemiologic Studies Depression Scale score of ≥19.

^f^Defined as a Center for Epidemiologic Studies Depression Scale score of <19.

^g^P value for interaction between sex and adipokines with depressive symptoms was assessed using the likelihood ratio test.

In multivariable adjusted models, higher ghrelin levels were associated with increased odds for depressive symptoms in women (*P*_*trend*_ = 0.02) (Table 3). The multivariable adjusted ORs (95% CIs) of depressive symptoms for the lowest through highest tertiles of ghrelin levels were 1.00 (reference), 1.71 (0.76-3.86), and 2.69 (1.16-6.28), respectively. Similarly, when another cutoff value for the definition of depressive symptoms (CES-D ≥19) was used, the ORs (95% CIs) for lowest through highest tertiles of ghrelin were 1.00 (reference), 1.45 (0.60-3.49), and 2.76 (1.71-5.38), respectively, in women (*P*_*trend*_ = 0.03). In men, ghrelin levels were not associated with depressive symptoms.

## Discussion

In an apparently healthy Japanese working population, women with higher ghrelin levels had higher odds for depressive symptoms than those with lower ghrelin levels, whereas women in the upper two-thirds of leptin levels had lower odds for depressive symptoms than those in the lowest third. In men, neither leptin nor ghrelin was associated with depressive symptoms. To our knowledge, this is the first study among Asians to examine the association between leptin and ghrelin levels and depressive status in the general population.

Our finding regarding leptin is in line with a previous clinical study [31], wherein leptin levels were significantly lower in female depressed patients than healthy controls, but the association was null in men. The present findings also concur with another study among suicide attempters [32], reporting that depressed women had lower leptin levels than non-depressed women, whereas no significant association was observed in men. As observed previously [31, 32], we also found that women, as compared with men, had higher levels of leptin. Because we found no reduction in the odds for men in the highest tertile of leptin (a level comparable to women in the middle tertile of leptin showing a reduction in the odds for depression), the gender difference in leptin levels may not account for the null finding in men. Alternatively, there may be a gender difference in the relationship between leptin and depression.

The finding of fewer depressive symptoms in women with higher levels of leptin is at odds with the results of two previous population-based studies. In a previous US study [42], leptin was not associated with depression in women, but higher leptin was associated with increased risk of depression in men with high visceral fat. In an Australian study [41], women with a lifetime history of depression had elevated levels of leptin, and leptin levels predicted the subsequent development of de novo major depressive disorder over five years of follow-up, independently of BMI. Given that obesity is associated with inflammation [53], which has been linked to both the risk for depression [54] and the development of leptin resistance [55], the positive association observed in these Western studies may be ascribed to a higher BMI of the participants. In a Japanese population, which has on average much lower BMI than Westerners, the association between leptin and mood disorder may be less likely to be influenced by such inflammation-depression pathway.

On the basis of contradictory data among epidemiological studies on the relationship between leptin and depressive symptoms, Lu et al. [10] suggested that both leptin insufficiency and leptin resistance might contribute to alterations of affective status. This could explain, at least in part, the non-linear relationship between leptin and depressive symptoms in the present study. We found no further reduction in the odds for depressive symptoms among women in the highest tertile of leptin, who had a higher mean BMI than those in the middle tertile of leptin, suggesting that reduced leptin sensitivity may mitigate any possible protective effects of leptin on depression in this group.

Ghrelin levels were significantly and positively associated with depressive symptoms among women in the present study. At variance with our finding, a clinical study reported that ghrelin level was not associated with depressive symptoms in women [30]. Likewise, other clinical studies found no significant difference in ghrelin levels between patients with major depression and healthy controls [27, 37, 38]. Another study reported significantly lower levels of ghrelin in depressive patients than in healthy controls [39]. However, in another study, suicide attempters had significantly higher levels of ghrelin than healthy controls [40]. Such inconsistency among clinical studies may be ascribed to the influence of depression on ghrelin levels or methodological shortcomings, such as small sample size and the lack of control for potential confounders including BMI and lifestyle factors. Population-based studies are needed to explore the association between ghrelin and depression.

A similar association between leptin, ghrelin, and depressive symptoms was observed when the presence of depressive symptoms was defined by two different cutoffs. Although the cutoff for CES-D ≥ 16 has been widely recommended, the cut off CES-D ≥ 19 has been recommended based on a validation study among Japanese workers [48]. Such a higher cutoff is reasonable for Japanese given that Japanese tend to express their positive emotion in a modest manner, potentially leading to a higher score on depression scales [56]. The consistency in relationships between leptin, ghrelin, and depressive symptoms using two different cutoffs of CES-D in defining depressive symptoms in our study strengthens our findings.

Our findings linking depression with higher level of leptin and lower levels of ghrelin seems reasonable given that leptin treatment improves monoamine neurotransmission [18, 19] and lessens HPA axis [21, 22], whereas ghrelin has opposite effects [24–26]. Because depression often results in profound alterations in dietary intakes, with either increased or decreased appetite, the observed differences in leptin and ghrelin levels between those with and without depression symptoms may simply reflect such change in dietary habit. However, this is a less plausible explanation for the present finding, as we found no significant differences in energy intake and BMI in subjects with depressive symptoms compared to those without.

Major strengths of the present study include the high study participation rate (94%), the exclusion of participants with history of chronic diseases, including type 2 diabetes, cardiovascular disease, and cancer, which may alter the levels of leptin or ghrelin, and the adjustment for potential cofounding variables. Despite these strengths, our study had several limitations that warrant mention. Firstly, data derived from a cross-sectional study cannot shed light on the direction of the relationship and it is not possible to determine causality. Secondly, ghrelin and leptin were measured only a single time point, which limits the accuracy of the measurement. However, Lee et al. [57] showed that adipokine levels measured in a single sample had a high correlation with the mean of the remaining three seasonal samples. Thirdly, because of the relatively small sample, statistical power might not have been sufficient to detect a moderate association. Fourthly, our study population was apparently healthy population and hence symptoms of depression in our sample were less than one would observe in clinical populations of patients with depression. Finally, our study was conducted in selected municipal offices, thus the findings may not be generalizable to the whole population of Japanese adults.

## Conclusions

In conclusion, this study show that higher ghrelin levels are associated with increased prevalence of depressive symptoms in women. Longitudinal studies with large sample size, assessments of serum leptin and ghrelin at multiple times point, and follow-up assessments for clinical depression are needed to confirm the present findings.
